# The power of the left atrioventricular coupling index in cardiovascular disease

**DOI:** 10.3389/fcvm.2025.1567856

**Published:** 2025-04-10

**Authors:** Xu Liu, Jing Wang, Yan Tong, Shuai Wang

**Affiliations:** ^1^ Department of Cardiology, The Second Affiliated Hospital of Shenyang Medical College, Shengyang, Liaoning, China; ^2^ Department of Social Services, Shengjing Hospital Affiliated to China Medical University, Shengyang, Liaoning, China

**Keywords:** left atrioventricular coupling index, cardiac function assessment, cardiovascular disease prognosis, cardiac imaging, heart failure prediction, atrial fibrillation

## Abstract

The left atrioventricular coupling index (LACI) has emerged as a novel and transformative biomarker in cardiovascular research, addressing long-standing limitations in traditional cardiac function assessments. By quantifying the ratio of left atrial to left ventricular end-diastolic volumes, LACI offers unprecedented prognostic insights into a wide range of cardiovascular diseases, including atrial fibrillation, heart failure, and myocardial infarction, as well as other conditions such as hypertension and cardiomyopathies. Recent evidence highlights its unique ability to integrate atrial and ventricular dynamics, offering a more comprehensive perspective on cardiac health and disease progression. This review synthesizes the latest advancements in LACI research, elucidates its underlying pathophysiological mechanisms, and explores its expanding clinical applications as a pivotal tool for risk stratification, precision diagnostics, and personalized therapy in cardiovascular medicine.

## 1 Introduction

Cardiovascular disease remains the leading cause of global morbidity and mortality, with its prevalence projected to increase by 90.0%, and related deaths expected to reach 35.6 million annually by 2050 (1). This underscores the critical need for innovative tools to enhance early detection and improve risk stratification. Conventional measures of cardiac function, such as left ventricular (LV) ejection fraction (LVEF) and global longitudinal strain, have long been foundational in clinical practice for assessing ventricular performance (2). Similarly, indices reflecting left atrial (LA) function, including LA function index and atrial strain, have demonstrated their value in predicting outcomes such as atrial fibrillation (AF) and heart failure (HF) (3–5). However, these conventional paradigms primarily assess either the atrium or ventricle independently and fail to account for the dynamic interaction between these chambers—an interaction that is central to normal cardiac physiology and pathophysiology.

In recent years, the left atrioventricular coupling index (LACI) has emerged as a novel parameter designed to address this limitation. By quantifying the ratio of LA to LV end-diastolic volumes during the mitral valve closure phase, LACI offers a more integrative assessment of atrial-ventricular interplay (6). This unique biomarker bridges traditional gaps in cardiac evaluation, providing insights into both atrial and ventricular dysfunction within the same framework. Furthermore, LACI demonstrates robust prognostic utility across a wide spectrum of cardiovascular conditions, including AF (7), HF (8), acute myocardial infarction(AMI) (9), and beyond. Its versatility is further enhanced by its compatibility with multiple imaging modalities, such as cardiac magnetic resonance imaging (CMR), computed tomography (CT), and transthoracic echocardiography, ensuring a broad range of clinical applications.

This review consolidates recent scientific advancements related to LACI, critically evaluating its emerging role in cardiovascular medicine. It provides an overview of the methodologies employed to measure LACI across varying imaging platforms, explores its association with key cardiovascular conditions, and discusses its potential to enhance diagnostic precision and prognostic accuracy. By synthesizing these findings, this review aims to support further advancement of LACI as a clinically invaluable tool for improving the management and risk stratification of patients with cardiovascular diseases.

## 2 Physiological and hemodynamic foundations of LA—LV coupling

**Figure 1:**
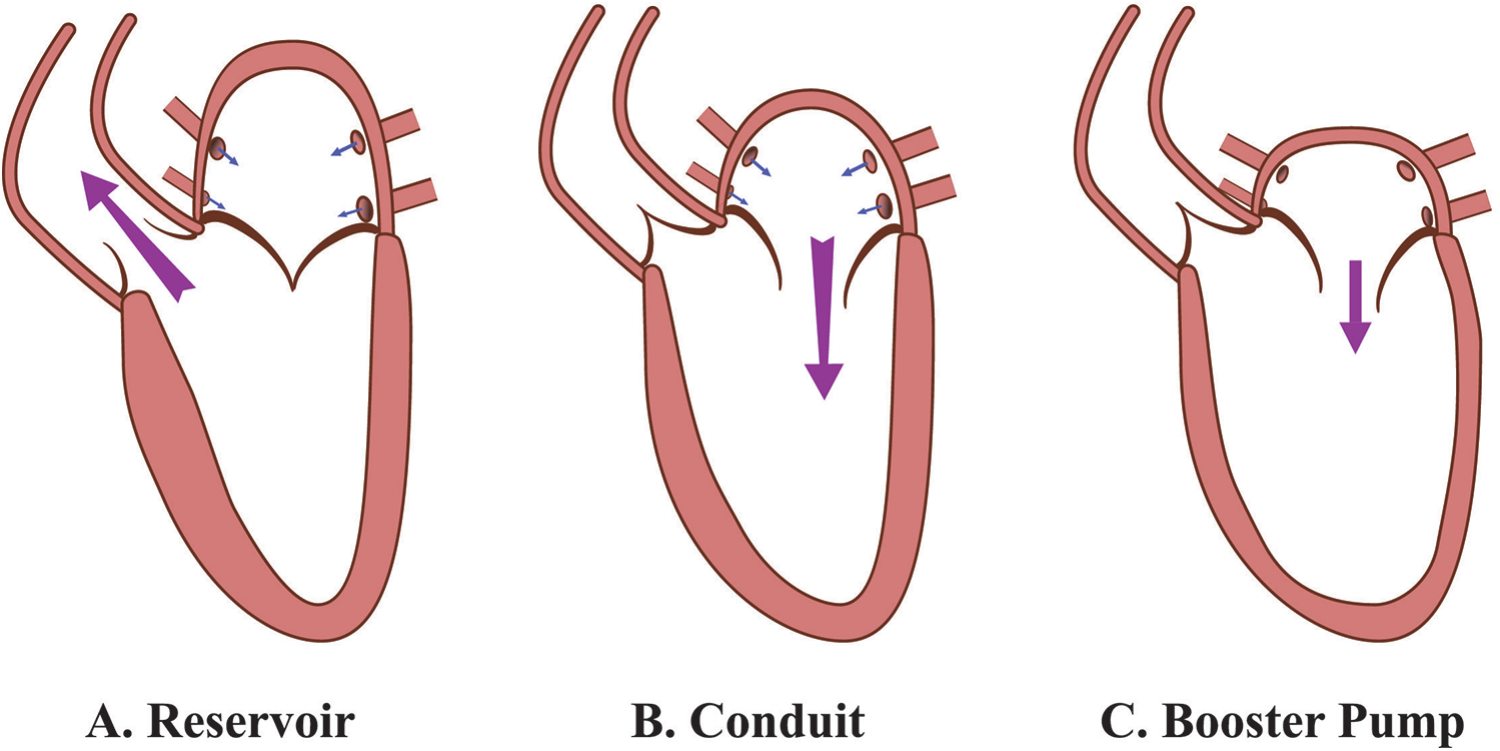
Functional roles of the LA during the cardiac cycle. **(A)** Reservoir phase: The LA serves as a reservoir, receiving blood from the pulmonary veins while the mitral valve is closed. During this phase, the LA expands to its maximal volume, while the LV ejects blood into the aorta through the open aortic valve. **(B)** Conduit phase: The mitral valve opens, and the LA functions as a passive conduit, allowing blood to flow from the pulmonary veins through the LA into the LV. This phase leads to LV expansion and filling, with minimal active involvement from the LA. **(C)** Booster pump phase: The LA contracts actively, functioning as a booster pump to enhance LV filling. This active contraction increases LA emptying volume, optimizing LV preload and priming the LV for systole. LA, left atrium; LV, left ventricle.

The LA plays a critical role in the cardiac cycle, fulfilling three distinct functions during different phases: the reservoir phase during ventricular systole, the conduit phase in early diastole, and the booster pump phase in late diastole (10). During ventricular systole, the downward displacement of the mitral annulus, driven by LV contraction, influences the compliance and relaxation of the LA, thereby determining its reservoir capacity. The energy stored in the LA during systole is subsequently released upon mitral valve opening, significantly contributing to an adequate LV stroke volume (11). Conversely, the conduit function of the LA is closely linked to LV diastolic properties, including the suction force generated during LV relaxation and the stiffness of the LV chamber. Meanwhile, the booster pump function of the LA depends on its contractile capacity, as well as the compliance and pressure of the LV at end-diastole (12). This process is further illustrated in Figure 1. Notably, in healthy individuals, the absence of atrial contraction—as observed in AF—can reduce LV stroke volume by approximately 20% to 30% (13).

Emerging evidence suggests that minimum LA volume serves as a more sensitive indicator of LV end-diastolic pressure compared to maximum LA volume (6). This can be attributed to the fact that the LA is exposed to LV pressure throughout diastole while the mitral valve remains open. Minimum LA volume has been shown to correlate more closely with invasive LA pressure measurements than maximum LA volume, thereby providing a more accurate reflection of LV filling pressures and elevated pulmonary capillary wedge pressure. This offers superior prognostic value in comparison to maximum LA volume (14–16).

**Figure 2:**
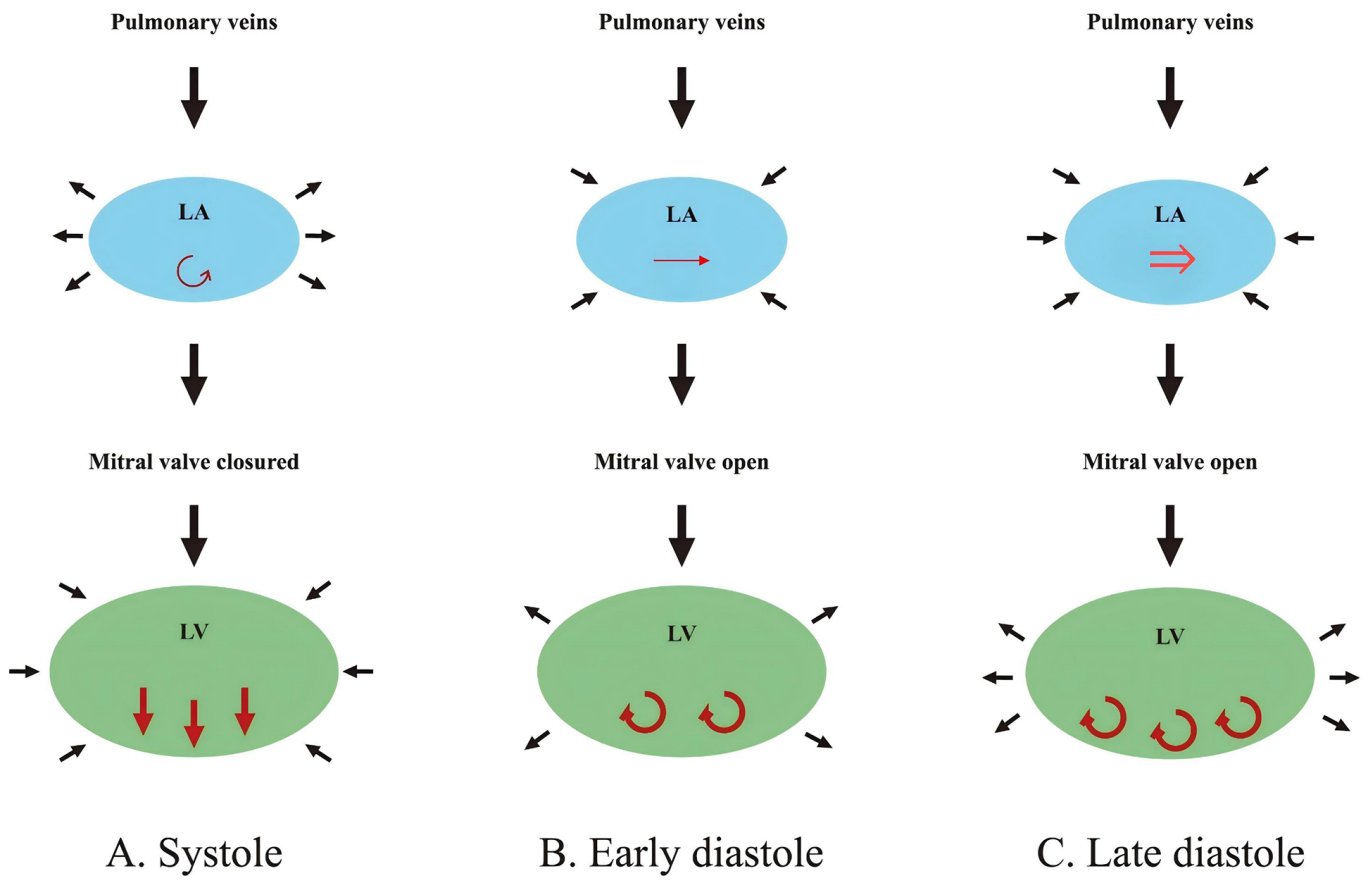
Hemodynamic changes in the LA and LV during different phases of the cardiac cycle. **(A)** Systole: The LA acts as a reservoir, receiving blood from the pulmonary veins while a weak vortex (↺, red rotating arrow) forms parallel to the closed mitral valve. The LV contracts, ejecting blood toward the aorta (↓↓↓, red downward arrows; aorta not shown), leading to LA expansion and LV volume reduction. **(B)** Early diastole: The mitral valve opens, allowing passive blood flow (→, red horizontal arrow) from the LA into the LV. A strong vortex (↻↻, red rotating arrows) forms in the LV, aiding its expansion, while LA volume decreases slightly. **(C)** Late diastole: The LA contracts actively, functioning as a booster pump to enhance LV filling (⇒, red double horizontal arrow). The LV vortex strengthens (↻↻↻, red rotating arrows), and LV volume reaches its maximum, while LA volume decreases further. LA, left atrium; LV, left ventricle.

From a hemodynamic perspective, diastole begins with the opening of the mitral valve, allowing blood to flow from the LA to the LV. At this point, rotational flow within the LA dissipates, giving way to the formation of vortex flow in the LV. This vortex flow, initially stronger than the preceding rotational flow in the LA during early diastole, generates kinetic energy that facilitates LV filling by increasing its diastolic volume. During late diastole, the vortex flow optimizes blood flow from the LA toward the LV outflow tract, aiding in stretching cardiomyocytes to enhance subsequent ventricular contraction. These interactions are critical for maintaining optimal preload adaptation and ensuring effective ventricular performance (17). A comprehensive depiction of this process is shown in Figure 2. Together, the physiological and hemodynamic interplay between the LA and LV highlights the central role of LA-LV coupling in cardiac function.

## 3 LACI measurement techniques

The LACI can be measured using multiple cardiac imaging modalities, each with distinct advantages and limitations. This section focuses on three common methods—CMR, echocardiography, and CT—for assessing LACI.

### 3.1 CMR: gold standard for precise LACI measurement

**Figure 3:**
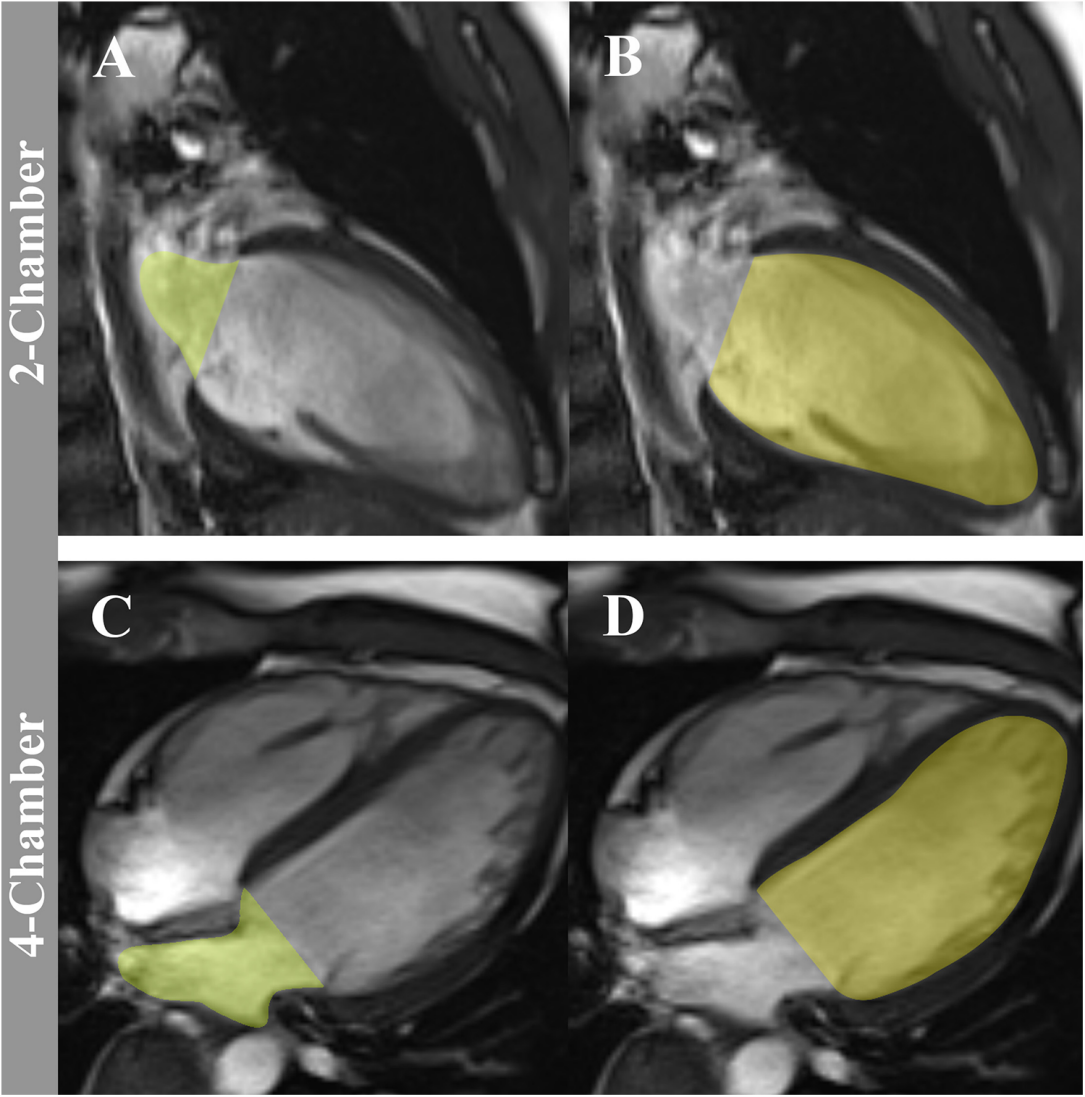
The method to assess LACI by CMR. LACI is calculated as the ratio of LA end-diastolic volume to LV end-diastolic volume. LA end-diastolic volume (green volume) is shown in the 2-chamber panel **(A)** and 4-chamber panel **(C)** views during end-diastole. LV end-diastolic volume (yellow volume) is shown in the 2-chamber panel **(B)** and 4-chamber panel **(D)** views. LACI, left atrioventricular coupling index; CMR, cardiovascular magnetic resonance; LA, left atrium; LV, left ventricle.

CMR is widely recognized as the gold standard for evaluating cardiac structure and function, primarily due to its ability to precisely quantify LA and LV volumes (18). The LACI is derived by calculating the ratio of the LA volume to the LV end-diastolic volume, with both parameters meticulously measured via CMR. During end-diastole, high-resolution short-axis cine images are obtained, and LV end-diastolic volume is measured using cardiac image modeler software. The LA volume is determined using the area-length method from 2- and 4-chamber views, with the biplane calculation performed using the following formula: LA volume = (0.848*Area^2ch^*Area^4ch^)/[(LA Length^2ch^ + Length^4ch^)]/2) (6, 19). It is noteworthy that the majority of CMR-derived LACI measurements are based on the method described by Pezel et al. (6) (see Figure 3).

The distinct advantages of CMR include high spatial resolution, exceptional image reproducibility, and the absence of ionizing radiation. These features make CMR a highly reliable tool for diagnosing and monitoring cardiac pathologies. However, its use may be limited by certain constraints, including high costs, prolonged image acquisition times, and limited availability in routine clinical settings. Despite these challenges, CMR remains indispensable in research and specialized clinical scenarios, solidifying its status as the gold standard for LACI evaluation.

### 3.2 Echocardiography: the practical and ubiquitous alternative

**Figure 4:**
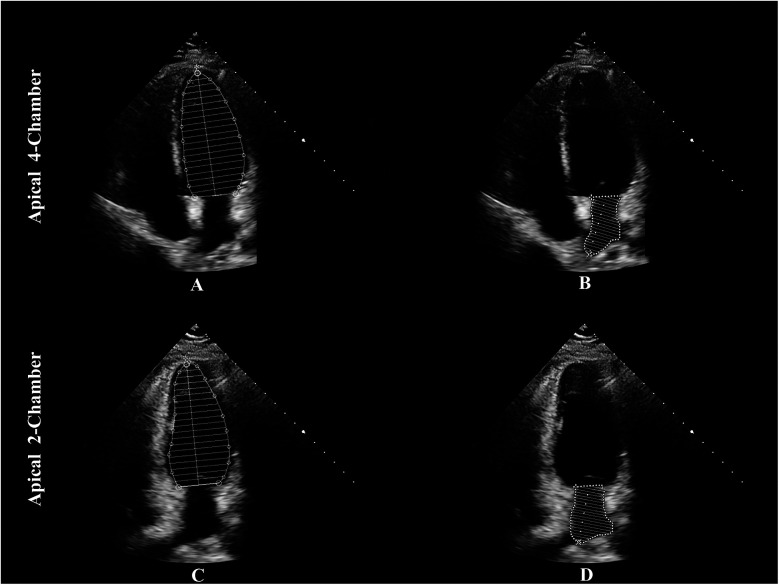
The method to assess LACI by echocardiography. LACI is calculated as the ratio of the minimum LA volume at end-diastole to the LV end-diastolic volume. The Simpson's biplane method is used to integrate measurements from apical two- and four-chamber views: panel **(A)** shows LVEDV in the apical 4-chamber view, panel **(B)** shows LAEDV in the apical 4-chamber view, panel **(C)** shows LVEDV in the apical 2-chamber view, and panel **(D)** shows LAEDV in the apical 2-chamber view. LACI, left atrioventricular coupling index; LA, left atrium; LV, left ventricle; LVEDV, left ventricular end-diastolic volume; LAEDV, left atrial end-diastolic volume.

Echocardiography remains the most widely used technique for assessing LACI, offering a non-invasive, cost-efficient, and readily available alternative to advanced imaging modalities such as CMR. In two-dimensional (2D) echocardiography, LACI is calculated as the ratio of the minimum LA volume at end-diastole to the LV end-diastolic volume, using the Simpson's biplane method, which integrates measurements from apical two- and four-chamber views (20) (see Figure 4).

However, 2D echocardiography has inherent limitations, including geometric assumptions, operator dependency, and variability in image acquisition. To address these challenges, three-dimensional (3D) echocardiography has emerged as a promising advancement, utilizing adaptive algorithms and speckle-tracking methods to provide fully automated LA and LV volume measurements. Unlike 2D echocardiography, 3D echocardiography eliminates the need for geometric modeling, thereby reducing inter-observer variability and shortening analysis times (21). Despite its advantages, echocardiographic results remain influenced by operator expertise and machine quality, highlighting the need for standardization across institutions to maximize its clinical utility.

### 3.3 CT: high-resolution imaging for LACI quantification

Another effective imaging modality for assessing LACI is CT, known for its high spatial resolution and rapid acquisition capability. Although its primary clinical application lies in the evaluation of coronary artery disease, CT can accurately quantify LA and LV volumes. Using electrocardiographic gating, the cardiac cycle is specifically analyzed to identify end-diastolic frames corresponding to the closure of the mitral valve, and subsequent multiplanar reconstruction yields high-fidelity images. The Simpson's method is applied to two- and four-chamber views to calculate the volumes of the LA and LV at end-diastole, which are then used for LACI determination (22).

Among the strengths of cardiac CT are its superior spatial resolution and rapid imaging times, which offer significant advantages in specialized settings. However, concerns about ionizing radiation exposure and risks associated with contrast media remain notable limitations. While not as widely used for functional cardiac assessment as echocardiography or CMR, CT continues to play a critical role in cardiovascular imaging.

In summary, CMR provides unparalleled precision and spatial resolution, establishing it as the gold standard for cardiac volume quantification, despite limitations related to cost and accessibility. Echocardiography remains the most widely used method due to its affordability and convenience, with advancements in 3D techniques significantly improving its accuracy and reducing variability. Cardiac CT, although less commonly utilized for functional assessment, offers rapid, high-resolution imaging and remains valuable in specific clinical scenarios. Ultimately, the choice of imaging modality should be guided by clinical priorities, resource availability, and investigative objectives.

## 4 Application of LACI in different clinical contexts

### 4.1 LACI and age

The structural and functional characteristics of the LA and LV exhibit significant variability across different age groups. In healthy individuals, advancing age is typically associated with an increase in the LA volume index and a decrease in the LV end-diastolic volume index (23, 24). While many studies have examined the LA or LV in isolation, relatively few have focused on their interdependent dynamics throughout the aging process. Using CMR, Germans et al. (25) investigated the effects of aging on the LA and LV volume indices by assessing the LA to LV volume ratio at both end-diastole and end-systole. Although they did not explicitly define the term “LACI”, their findings that this ratio increases with age set the groundwork for further exploration. Their findings revealed that the LA to LV volume ratio was notably higher in older individuals compared to younger cohorts, both at end-diastole (0.3 vs. 0.2) and end-systole (1.6 vs. 1.2; *P* < 0.001). Building on this, Takeuchi et al. (26) employed 3D echocardiography in a cohort of 313 healthy volunteers to assess the inverse parameter—the LV to LA volume ratio (LVLAVR). They observed significant age-related reductions in LVLAVR, particularly at end-diastole, underscoring the sensitivity of this metric to aging-associated changes in cardiac geometry.

These changes in LA and LV dimensions can be attributed partly to an elevated LV mass-to-volume ratio observed in elderly populations, which reduces ventricular compliance. This reduction in compliance triggers compensatory LA enlargement, helping to offset the increasing LA pressure (25). Such compensatory mechanisms illustrate the intricate interplay between the LA and LV within the context of age-related myocardial remodeling. Although Takeuchi et al. have provided critical reference values for LA-LV coupling indices across age groups, further large-scale, multicenter investigations are warranted. Validation of these findings in racially and ethnically diverse populations is essential to refine our understanding of age-induced LA and LV remodeling. Such studies hold the potential to enhance the accuracy of clinical assessments and improve individualized diagnostic approaches for patients from varied demographic backgrounds.

### 4.2 LACI and AF

AF is the most common arrhythmia encountered in clinical practice and is strongly associated with adverse outcomes, including HF, stroke, cognitive decline, vascular dementia, and reduced quality of life. Consequently, AF significantly increases patient mortality risk (27). Early identification of risk factors and improved predictive tools for AF are therefore essential to optimize management. Recent investigations have highlighted the potential role of the LACI in predicting AF incidence, recurrence, and its relationship with exercise capacity.

In the Multi-Ethnic Study of Atherosclerosis (MESA), LACI derived from CMR emerged as an independent predictor of AF. Baseline measurements of LACI were associated with an increased hazard for developing AF [hazard ratio [HR]: 1.81, 95% confidence interval [CI]: 1.57–2.01; *P* < 0.001], a relationship that persisted after 10 years of follow-up (HR: 1.69, 95% CI: 1.46–1.96; *P* < 0.001) (7). A multivariable model incorporating LACI demonstrated superior predictive power for incident AF over 10 years, outperforming the traditional CHARGE-AF (Cohort for Heart and Aging Research in Genomic Epidemiology-Atrial Fibrillation) model and combinations of individual LA or LV parameters with CHARGE-AF (7).

In hypertrophic cardiomyopathy (HCM) patients, LACI was shown to be a more robust predictor of new-onset AF compared to conventional LA parameters (20). Additionally, among patients with paroxysmal AF undergoing radiofrequency ablation, elevated LACI was significantly associated with post-procedural AF recurrence, underscoring its value as an independent marker for reduced LA contractile function and increased LA volume (28). Collectively, these findings highlight the clinical utility of LACI in AF management, emphasizing its applications in risk stratification, therapeutic decision-making, and long-term prognostication.

From a mechanistic perspective, adverse LA remodeling plays a central role in the initiation and maintenance of AF. This remodeling facilitates ectopic triggers and alters the reentrant arrhythmia circuits through changes in wavelength and conduction properties (29). However, the development of AF is not solely linked to structural and functional changes in the LA. LV diastolic dysfunction, which impairs LV compliance, also contributes to an imbalance between the two chambers. This dysfunction increases LA volume relative to LV volume at end-diastole, reflecting impaired LV compliance and reduced LA reserve function—factors that are independently predictive of AF (30). These findings suggest that AF arises not only from isolated abnormalities in LA or LV structure and function but also from a disruption of atrial-ventricular coupling. LACI, which integratively captures this coupling, provides a clearer depiction of the interplay between LA and LV function, making it a valuable tool for anticipating AF risk and optimizing clinical management strategies.

#### 4.2.1 Challenges in measuring LACI in AF

Obtaining accurate LACI measurements in patients with AF can be challenging due to beat-to-beat variability. To address these challenges, several strategies can be employed:

Using 3D Echocardiography: 3D echocardiography offers several advantages in accurately capturing chamber volumes, especially in AF patients. It reduces the impact of geometric assumptions by directly measuring cardiac volumes, provides higher accuracy and repeatability compared to 2D echocardiography (especially in cases of irregular LV shapes), and optimizes temporal resolution by capturing data at multiple time points throughout the cardiac cycle (31).

Averaging Multiple Cardiac Cycles: By averaging volume data across multiple cardiac cycles, the variability caused by irregular rhythms can be minimized, providing a more representative measure of LA and LV volumes.

Selecting Relatively Stable Cycles: Using electrocardiographic guidance, clinicians can identify and select relatively stable cardiac cycles for measurement. This involves choosing cycles with minimal beat-to-beat variability, thereby improving measurement consistency.

While these strategies show promise, they have not yet been specifically validated for LACI measurement in AF. Future studies are needed to validate and refine these methodologies for clinical practice.

### 4.3 LACI and HF

Impaired coupling between the LA and LV has emerged as a critical contributor to the pathogenesis of HF with preserved ejection fraction (HFpEF). Animal studies have highlighted a reduction in LA compliance, reflected in an upward and leftward shift of the LA end-diastolic pressure-volume relationship, providing mechanistic insight into this impairment (32). Clinically, evidence from the MESA underscores the importance of this relationship. Pezel et al. (8). followed 2,250 participants without a history of HF or cardiovascular disease for up to 10 years, finding that those who developed HF mostly retained preserved ejection fractions. The LACI was significantly higher in the HF group compared to the non-HF group (41.2 ± 12.1 vs. 26.1 ± 10.2; *P* < 0.001), enhancing the discriminative ability of HF risk prediction models.

Beyond risk prediction, LACI has demonstrated robust diagnostic and prognostic value in HFpEF. For instance, Backhaus et al. (33) found that LACI showed the highest diagnostic accuracy at rest, effectively distinguishing HFpEF patients from those with noncardiac dyspnea. In a separate cohort of 1,158 stable HF patients, LACI emerged as an independent predictor of all-cause mortality and HF-related hospitalizations, surpassing the predictive accuracy of the traditional grading system for diastolic dysfunction (net reclassification improvement = 0.150, *P* < 0.0001) (34). Among patients with HF with reduced ejection fractions (LVEF < 50%), Kasa et al. (35) found that elevated LACI was significantly associated with the composite outcome of all-cause mortality or HF hospitalization (adjusted HR: 1.77, *P* = 0.02). These findings underscore the versatility of LACI across various HF phenotypes as both a diagnostic tool and a prognostic measure.

With advancements in artificial intelligence (AI) technology, AI-automated measurement of LACI offers a novel approach for prognostic assessment in HF. Studies have shown that AI-measured LACI independently predicts the risk of HF hospitalization or cardiovascular death, demonstrating added prognostic value beyond traditional risk factors (36). This technology may enhance the efficiency and accuracy of LACI measurement, reinforcing its role in HF management.

From a pathophysiological standpoint, remodeling of the LA plays a pivotal role in the progression of HF, particularly in HFpEF. During the early stages of diastolic dysfunction, the LA undergoes adaptive structural and functional changes to sustain cardiac output. However, prolonged exposure to elevated LV filling pressures culminates in fibrosis, reduced compliance, and increased stiffness within the atrial myocardium, thereby driving a pathological rise in LACI (13, 37). Over time, these maladaptive responses contribute to atrial enlargement and uncoupling of LA-LV dynamics, even when the systolic function of the LV remains preserved (38, 39). By integrating changes in LA structure with the functional relationship between the two chambers, LACI provides a comprehensive metric for evaluating disease progression and tailoring therapeutic strategies across the spectrum of HF phenotypes.

### 4.4 LACI and AMI

Alterations in atrioventricular coupling have garnered attention as a key pathological feature in AMI. In a study by Liu et al. (9), AMI patients demonstrated significant impairment in LA function and LV deformation, accompanied by elevated LACI levels compared to healthy controls. These findings were consistent regardless of the presence of metabolic syndrome, highlighting the pervasive impact of AMI on atrial and ventricular mechanics.

The prognostic implications of LACI in post-AMI settings were further explored by Lange et al. (40), who assessed the relationship between CMR-derived LACI values and major adverse cardiovascular events (MACE) within 12 months after an AMI. Their results indicated that patients who experienced MACE exhibited significantly elevated LACI levels. These findings underscore the potential of LACI as a reliable marker for post-MI risk stratification and patient management.

However, the instability of patient conditions during the acute phase of AMI limits the application of CMR examinations; thus, these studies focused primarily on patients in a stable phase. Echocardiography, due to its convenience and non-invasiveness, shows promise as an alternative method for assessing LACI during the acute phase.

### 4.5 LACI and hypertension

Arterial hypertension increases LV afterload, subsequently elevating LV filling pressure and LA pressure (41). In hypertensive patients with LV hypertrophy, the LA exhibits reduced reservoir, conduit, and booster pump functions, accompanied by increased LA stiffness (42), reflecting impaired LA-LV coupling. These changes are closely linked to LV hypertrophy and dysfunction.

Recent studies have demonstrated that diabetic patients with coexisting hypertension exhibit significantly higher LACI levels compared to those with normal blood pressure (22.72 ± 15.01 vs. 17.40 ± 10.28; *P* = 0.049) (19). Moreover, hypertension itself has been identified as an independent risk factor for increased LACI (β = 0.05; *P* < 0.011). Excessive sodium intake, a key contributor to hypertension (43), significantly raises blood pressure (44) and is best measured using 24 h urinary sodium excretion, the gold standard for assessing sodium consumption (45). Supporting this, Yin et al. demonstrated that individuals with higher 24 h urinary sodium excretion exhibited significantly elevated LACI levels compared to those with lower sodium excretion (25.92 ± 15.45 vs. 18.73 ± 10.10; *P* < 0.001) (46).

Mechanistically, excessive sodium intake promotes myocardial proliferation, hypertrophy, and collagen deposition, leading to myocardial fibrosis and hypertrophy (47). These pathological changes reduce LA compliance (13) and impair LA-LV coupling (42), highlighting the significance of salt restriction in managing hypertension and mitigating cardiac dysfunction.

### 4.6 LACI and coronary microvascular dysfunction

Meta-analyses have established that coronary microvascular dysfunction (CMD) is linked to higher mortality and an increased risk of MACE (48). However, screening for CMD in clinical practice remains a significant challenge. A study by Tran et al. (49), involving 32 patients with HCM, found that patients with CMD exhibited significantly higher LACI compared to those without CMD (*P* = 0.03). Further multivariate logistic regression analysis identified LACI as an independent predictor of CMD.

Although the sample size in this study was limited, the findings suggest that LACI may serve as a valuable diagnostic marker for CMD in various clinical scenarios. Furthermore, CMD is often managed with medications such as beta-blockers and calcium channel blockers. Whether these treatments lead to improvements in LACI remains an open question, necessitating further research.

### 4.7 LACI and cardiomyopathies

In the context of dilated cardiomyopathy (DCM), Vîjîiac et al. (50) demonstrated that the LACI, right atrioventricular coupling index (RACI), and the combined atrioventricular coupling index (CACI), derived from both, can significantly predict adverse events as assessed by 3D echocardiography. Notably, CACI exhibited superior prognostic capabilities compared to traditional risk factors in multivariable Cox regression analysis. Similarly, in HCM, LACI has proven valuable in predicting the onset of AF (20) and CMD (49). Additionally, Wen et al. (51) highlighted LACI as an independent predictor of adverse clinical outcomes in HCM, demonstrating its potential to enhance risk stratification and predictive accuracy when integrated with traditional markers.

In early-stage Fabry disease (FD), 2D speckle-tracking echocardiography revealed that LACI was significantly elevated compared to healthy controls. It was positively correlated with age and E/e’ and negatively correlated with LA strain rate, highlighting its potential for early risk stratification (52). For light-chain amyloidosis cardiomyopathy (AL-CA), 3D echocardiography showed that LACI was independently associated with all-cause mortality and provided additional prognostic value beyond traditional staging models (53). Furthermore, Wang et al. (54) validated the prognostic utility of LACI in AL-CA patients using CMR, demonstrating its role as an independent predictor of all-cause mortality.

LACI demonstrates significant potential in the clinical management of cardiomyopathies, offering robust capabilities for prognosis assessment and risk stratification in DCM, HCM, FD, and AL-CA. It represents a critical tool for improving patient care in these conditions. Future research should explore the underlying mechanisms of LACI in various cardiomyopathies and its integration with other clinical parameters to optimize its application and enhance patient outcomes.

### 4.8 LACI and myocarditis

The application of LACI in the clinical management of myocarditis is still in an exploratory stage. In a study of patients with suspected myocarditis and preserved LVEF, CMR-assessed LACI showed no significant correlation with MACE (*P* = 0.847). However, LA conduit strain was significantly associated with MACE occurrence (*P* = 0.031) (55). This divergence may stem from the superior sensitivity of strain analysis in detecting subtle functional abnormalities before structural or volumetric changes manifest. Further research is needed to investigate the potential utility of LACI in assessing various subtypes and severities of myocarditis, as well as its integration with advanced imaging techniques and biomarkers for enhanced clinical decision-making.

### 4.9 LACI and aortic stenosis

Transcatheter aortic valve replacement (TAVR) has become a preferred treatment for symptomatic severe aortic stenosis, particularly in patients at high surgical risk or deemed ineligible for surgical aortic valve replacement (56). In a study by Zsarnoczay et al. (57), an AI-based fully automated assessment of LACI was shown to independently predict mortality in TAVR patients, including those with preserved LVEF. These findings highlight LACI's potential as a valuable prognostic marker in this specific clinical context.

## 5 LACI clinical thresholds

The LACI has demonstrated significant prognostic value across various cardiac conditions, including AF, HF, AMI, HCM, and AL-CA. However, comprehensive standardization remains a challenge.

### 5.1 Condition-specific LACI thresholds

Current studies define disease-specific thresholds tailored to distinct pathologies and patient cohorts. Table 1 summarizes key findings and their clinical implications:

**Table 1:** Disease-specific LACI thresholds and their clinical applications.

Condition	LACI cut-off	Key findings	Clinical Implications
AF	>30% (7), ≥ 40% (20)	Predicts incidence	Enables earlier detection, risk stratification, and more effective management of AF
	34.5% (28)	Recurrence post-ablation	
HF	>30% (8)	Predicts incidence	Stratifies risk for HF events and diastolic dysfunction.
	≥25% (36)	Hospitalization/mortality	
	>26% (34)	Identifies diastolic dysfunction	
AMI	34.7% (41)	Indicates high risk of MACE in AMI survivors	Guides post-AMI management to reduce cardiovascular risk
HCM	40.09% (52)	Enhances risk stratification for adverse outcomes	Refines decision-making for HCM risk management
AL-CA	≥57% (54), ≥49.3% (55)	All-cause mortality	Improve prognosis estimation in AL-CA patients
Aortic Stenosis (TAVR)	≥43.7% (58)	Predicts mortality post-TAVR.	Risk stratification for patients post-TAVR procedures

LACI, left atrioventricular coupling index; AF, atrial fibrillation; HF, heart failure; AMI, acute myocardial infarction; MACE, major adverse cardiovascular events; HCM, hypertrophic cardiomyopathy; AL-CA, amyloidosis cardiomyopathy; TAVR, transcatheter aortic valve replacement.

These condition-specific thresholds highlight LACI's clinical utility in risk stratification and therapeutic decision-making.

### 5.2 Normal range and standardization challenges

Defining a universally accepted normal range for LACI is essential for distinguishing disease thresholds from physiological norms. Evidence from the MESA study indicates that mean LACI values among individuals without baseline cardiovascular disease typically range from 16.4 ± 7.5% to 17.7 ± 9.1% (6, 8, 58, 59), averaging approximately 17 ± 8%.

#### 5.2.1 Key challenges include

Inter-study Variability: Differences in cohort characteristics and imaging protocols create inconsistencies in reported values.

Demographic Influences: Factors like age, sex, and ethnicity affect LACI (59); for example, older adults may exhibit naturally lower values due to altered cardiac physiology.

Imaging Modalities and Protocols: Protocol inconsistencies across echocardiography, MRI, and CT imaging necessitate standardization.

While the MESA study offers a valuable baseline, further large-scale, multi-ethnic research is crucial to refine and standardize normal values.

## 6 Future directions

To further define the clinical utility of the LACI as a prognostic biomarker in cardiovascular medicine, several key research directions should be prioritized. One crucial focus is the validation of normal and pathological LACI ranges. Large, multi-center studies are needed to establish universally applicable reference values that account for the influence of demographic factors such as age, sex, and ethnicity. Understanding these variations will aid in distinguishing physiological norms from pathological deviations, thereby enhancing the clinical interpretation of LACI across diverse populations.

Another promising avenue is the development of AI-driven automated measurement techniques. Advanced machine learning algorithms have the potential to significantly improve the accuracy and consistency of LACI quantification, minimizing inter- and intra-observer variability. These AI tools can streamline the analysis of large datasets, making LACI assessments more efficient and precise in both clinical and research settings. By enabling standardized, high-precision measurements, AI could play a pivotal role in accelerating the adoption of LACI as a routinely utilized marker in cardiovascular care.

Additionally, standardization of LACI calculation across imaging modalities is a critical step toward broader clinical application. Currently, LACI can be derived from techniques such as echocardiography, CMR, and CT, but variability in measurement protocols and image acquisition methods can lead to inconsistencies in reported values. Establishing consensus on imaging protocols and thresholds, particularly for resting LACI, will ensure greater reproducibility and comparability of data. Collaborative efforts among professional societies, imaging experts, and researchers are essential to harmonize these practices and fully validate LACI as a reliable prognostic marker across various cardiovascular conditions.

## 7 Conclusion

In summary, the LACI has gained recognition as an innovative prognostic marker in cardiovascular medicine, offering a unique perspective by integrating LA and LV dynamics. Built upon a solid physiological and hemodynamic framework, LACI has shown significant potential in enhancing risk prediction and clinical decision-making across a diverse spectrum of conditions, including AF, HF, AMI, hypertension, cardiomyopathies, coronary microvascular dysfunction, myocarditis, and valvular disorders. Its broad applicability underscores its value in refining patient management and guiding treatment strategies. Future advancements will rely on the development of AI-driven tools to enhance measurement accuracy and efficiency, the standardization of imaging protocols across modalities, and the establishment of universally accepted thresholds through large, multi-center studies. Integrating LACI into multimodal risk models could further solidify its role in precision care, supporting individualized therapeutic planning and advancing cardiovascular medicine.

## References

[1] Chong B Jayabaskaran J Jauhari SM Chan SP Goh R Kueh MTW Global burden of cardiovascular diseases: projections from 2025 to 2050. Eur J Prev Cardiol. (2024):zwae281. 10.1093/eurjpc/zwae281 39270739

[2] Potter E Marwick TH. Assessment of left ventricular function by echocardiography: the case for routinely adding global longitudinal strain to ejection fraction. JACC Cardiovasc Imaging. (2018) 11(2 Pt 1):260–74. 10.1016/j.jcmg.2017.11.017 29413646

[3] Debonnaire P Joyce E Hiemstra Y Mertens BJ Atsma DE Schalij MJ Left atrial size and function in hypertrophic cardiomyopathy patients and risk of new-onset atrial fibrillation. Circ Arrhythm Electrophysiol. (2017) 10(2):e004052. 10.1161/circep.116.004052 28183843

[4] Sargento L Vicente Simões A Longo S Lousada N Palma Dos Reis R. Left atrial function index predicts long-term survival in stable outpatients with systolic heart failure. Eur Heart J Cardiovasc Imaging. (2017) 18(2):119–27. 10.1093/ehjci/jew196 27679598

[5] Sardana M Lessard D Tsao CW Parikh NI Barton BA Nah G Association of left atrial function index with atrial fibrillation and cardiovascular disease: the framingham offspring study. J Am Heart Assoc. (2018) 7(7):e008435. 10.1161/jaha.117.008435 29602764 PMC5907604

[6] Pezel T Venkatesh BA De Vasconcellos HD Kato Y Shabani M Xie E Left atrioventricular coupling index as a prognostic marker of cardiovascular events: the MESA study. Hypertension. (2021) 78(3):661–71. 10.1161/hypertensionaha.121.17339 34225471 PMC8363553

[7] Pezel T Ambale-Venkatesh B Quinaglia T Heckbert SR Kato Y de Vasconcellos HD Change in left atrioventricular coupling Index to predict incident atrial fibrillation: the multi-ethnic study of atherosclerosis (MESA). Radiology. (2022) 303(2):317–26. 10.1148/radiol.210315 35191736 PMC9081516

[8] Pezel T Ambale Venkatesh B Kato Y De Vasconcellos HD Heckbert SR Wu CO Left atrioventricular coupling Index to predict incident heart failure: the multi-ethnic study of atherosclerosis. Front Cardiovasc Med. (2021) 8:704611. 10.3389/fcvm.2021.704611 34540915 PMC8442844

[9] Liu J Li Y Peng LQ Gao Y Shi K Qian WL Effect of metabolic syndrome on left atrial and left ventricular deformation and atrioventricular interactions in patients with myocardial infarction. J Magn Reson Imaging. (2025) 61(1):235–47. 10.1002/jmri.29406 38682602

[10] Barbier P Solomon SB Schiller NB Glantz SA. Left atrial relaxation and left ventricular systolic function determine left atrial reservoir function. Circulation. (1999) 100(4):427–36. 10.1161/01.cir.100.4.427 10421605

[11] Mandoli GE Sisti N Mondillo S Cameli M. Left atrial strain in left ventricular diastolic dysfunction: have we finally found the missing piece of the puzzle? Heart Fail Rev. (2020) 25(3):409–17. 10.1007/s10741-019-09889-9 31773504

[12] Toma Y Matsuda Y Moritani K Ogawa H Matsuzaki M Kusukawa R. Left atrial filling in normal human subjects: relation between left atrial contraction and left atrial early filling. Cardiovasc Res. (1987) 21(4):255–9. 10.1093/cvr/21.4.255 3652092

[13] Thomas L Marwick TH Popescu BA Donal E Badano LP. Left atrial structure and function, and left ventricular diastolic dysfunction: JACC state-of-the-art review. J Am Coll Cardiol. (2019) 73(15):1961–77. 10.1016/j.jacc.2019.01.059 31000000

[14] Russo C Jin Z Homma S Rundek T Elkind MS Sacco RL Left atrial minimum volume and reservoir function as correlates of left ventricular diastolic function: impact of left ventricular systolic function. Heart (Br Cardiac Soc). (2012) 98(10):813–20. 10.1136/heartjnl-2011-301388 PMC3392716 22543839

[15] Wu VC Takeuchi M Kuwaki H Iwataki M Nagata Y Otani K Prognostic value of LA volumes assessed by transthoracic 3D echocardiography: comparison with 2D echocardiography. JACC Cardiovasc Imaging. (2013) 6(10):1025–35. 10.1016/j.jcmg.2013.08.002 24011776

[16] Hedberg P Selmeryd J Leppert J Henriksen E. Left atrial minimum volume is more strongly associated with N-terminal pro-B-type natriuretic peptide than the left atrial maximum volume in a community-based sample. Int J Cardiovasc Imaging. (2016) 32(3):417–25. 10.1007/s10554-015-0800-1 26498654 PMC4751167

[17] Sengupta PP Narula J. À LA mode atrioventricular mechanical coupling. JACC Cardiovasc Imaging. (2014) 7(1):109–11. 10.1016/j.jcmg.2013.12.001 24433719

[18] Petersen SE Aung N Sanghvi MM Zemrak F Fung K Paiva JM Reference ranges for cardiac structure and function using cardiovascular magnetic resonance (CMR) in Caucasians from the UK Biobank population cohort. J Cardiovasc Magn Reson. (2017) 19(1):18. 10.1186/s12968-017-0327-9 28178995 PMC5304550

[19] Shi R Jiang YN Qian WL Guo YK Gao Y Shen LT Assessment of left atrioventricular coupling and left atrial function impairment in diabetes with and without hypertension using CMR feature tracking. Cardiovasc Diabetol. (2023) 22(1):295. 10.1186/s12933-023-01997-z 37904206 PMC10617180

[20] Meucci MC Fortuni F Galloo X Bootsma M Crea F Bax JJ Left atrioventricular coupling index in hypertrophic cardiomyopathy and risk of new-onset atrial fibrillation. Int J Cardiol. (2022) 363:87–93. 10.1016/j.ijcard.2022.06.017 35716931

[21] Takeuchi M Nabeshima Y Kitano T Negishi K. Prognostic value of the left ventricular—left atrial volume ratio assessed using three-dimensional echocardiography with fully automated analytical software. J Cardiol. (2021) 78(5):406–12. 10.1016/j.jjcc.2021.05.004 34088561

[22] Pezel T Dillinger JG Toupin S Mirailles R Logeart D Cohen-Solal A Left atrioventricular coupling index assessed using cardiac CT as a prognostic marker of cardiovascular death. Diagn Interv Imaging. (2023) 104(12):594–604. 10.1016/j.diii.2023.06.009 37353467

[23] Fuchs A Mejdahl MR Kühl JT Stisen ZR Nilsson EJ Køber LV Normal values of left ventricular mass and cardiac chamber volumes assessed by 320-detector computed tomography angiography in the Copenhagen general population study. Eur Heart J Cardiovasc Imaging. (2016) 17(9):1009–17. 10.1093/ehjci/jev337 26758412

[24] Eriksen-Volnes T Grue JF Hellum Olaisen S Letnes JM Nes B Løvstakken L Normalized echocardiographic values from guideline-directed dedicated views for cardiac dimensions and left ventricular function. JACC Cardiovasc Imaging. (2023) 16(12):1501–15. 10.1016/j.jcmg.2022.12.020 36881415

[25] Germans T Götte MJ Nijveldt R Spreeuwenberg MD Beek AM Bronzwaer JG Effects of aging on left atrioventricular coupling and left ventricular filling assessed using cardiac magnetic resonance imaging in healthy subjects. Am J Cardiol. (2007) 100(1):122–7. 10.1016/j.amjcard.2007.02.060 17599453

[26] Takeuchi M Kitano T Nabeshima Y Otsuji Y Otani K. Left ventricular and left atrial volume ratio assessed by three-dimensional echocardiography: novel indices for evaluating age-related change in left heart chamber size. Physiol Rep. (2019) 7(23):e14300. 10.14814/phy2.14300 31814325 PMC6900493

[27] Chung MK Refaat M Shen WK Kutyifa V Cha YM Di Biase L Atrial fibrillation: JACC council perspectives. J Am Coll Cardiol. (2020) 75(14):1689–713. 10.1016/j.jacc.2020.02.025 32273035

[28] Li A Zhang M Ning B. Predictive value of the left atrioventricular coupling index for recurrence after radiofrequency ablation of paroxysmal atrial fibrillation. J Cardiothorac Surg. (2024) 19(1):552. 10.1186/s13019-024-03070-6 39354511 PMC11443840

[29] Nattel S Burstein B Dobrev D. Atrial remodeling and atrial fibrillation: mechanisms and implications. Circ Arrhythm Electrophysiol. (2008) 1(1):62–73. 10.1161/circep.107.754564 19808395

[30] Abhayaratna WP Fatema K Barnes ME Seward JB Gersh BJ Bailey KR Left atrial reservoir function as a potent marker for first atrial fibrillation or flutter in persons > or=65 years of age. Am J Cardiol. (2008) 101(11):1626–9. 10.1016/j.amjcard.2008.01.051 18489941

[31] Lang RM Badano LP Mor-Avi V Afilalo J Armstrong A Ernande L Recommendations for cardiac chamber quantification by echocardiography in adults: an update from the American society of echocardiography and the European association of cardiovascular imaging. Eur Heart J Cardiovasc Imaging. (2015) 16(3):233–70. 10.1093/ehjci/jev014 25712077

[32] Zakeri R Moulay G Chai Q Ogut O Hussain S Takahama H Left atrial remodeling and atrioventricular coupling in a canine model of early heart failure with preserved ejection fraction. Circ Heart Fail. (2016) 9(10):e003238. 10.1161/circheartfailure.115.003238 27758811 PMC5082983

[33] Backhaus SJ Lange T Schulz A Evertz R Frey SM Hasenfuß G Cardiovascular magnetic resonance rest and exercise-stress left atrioventricular coupling index to detect diastolic dysfunction. Am J Physiol Heart Circ Physiol. (2023) 324(5):H686–h95. 10.1152/ajpheart.00081.2023 36897745

[34] Fortuni F Biagioli P Myagmardorj R Mengoni A Chua AP Zuchi C Left atrioventricular coupling index: a novel diastolic parameter to refine prognosis in heart failure. J Am Soc Echocardiogr. (2024) 37(11):1038–46. 10.1016/j.echo.2024.06.013 38950757

[35] Kasa G Teis A De Raffele M Cediel G Juncà G Lupón J Prognostic value of left atrioventricular coupling index in heart failure. Eur Heart J Cardiovasc Imaging. (2025) 26(4):610–7. 10.1093/ehjci/jeaf010 39792882

[36] Pezel T Garot P Toupin S Sanguineti F Hovasse T Unterseeh T AI-based fully automated left atrioventricular coupling index as a prognostic marker in patients undergoing stress CMR. JACC Cardiovasc Imaging. (2023) 16(10):1288–302. 10.1016/j.jcmg.2023.02.015 37052568

[37] Santos AB Kraigher-Krainer E Gupta DK Claggett B Zile MR Pieske B Impaired left atrial function in heart failure with preserved ejection fraction. Eur J Heart Fail. (2014) 16(10):1096–103. 10.1002/ejhf.147 25138249 PMC5535768

[38] Payne RM Stone HL Engelken EJ. Atrial function during volume loading. J Appl Physiol. (1971) 31(3):326–31. 10.1152/jappl.1971.31.3.326 5111850

[39] Cameli M Pastore MC Mandoli GE. Left atrial strain: a key element for the evaluation of patients with HFpEF. Int J Cardiol. (2021) 323:197–8. 10.1016/j.ijcard.2020.09.078 33027680

[40] Lange T Backhaus SJ Schulz A Evertz R Kowallick JT Bigalke B Cardiovascular magnetic resonance-derived left atrioventricular coupling index and major adverse cardiac events in patients following acute myocardial infarction. J Cardiovasc Magn Reson. (2023) 25(1):24. 10.1186/s12968-023-00929-w 37046343 PMC10099819

[41] Alsharari R Oxborough D Lip GYH Shantsila A. Myocardial strain imaging in resistant hypertension. Curr Hypertens Rep. (2021) 23(5):24. 10.1007/s11906-021-01148-3 33950321 PMC8099817

[42] Soullier C Niamkey JT Ricci JE Messner-Pellenc P Brunet X Schuster I. Hypertensive patients with left ventricular hypertrophy have global left atrial dysfunction and impaired atrio-ventricular coupling. J Hypertens. (2016) 34(8):1615–20. 10.1097/hjh.0000000000000971 27219488

[43] Mente A O'Donnell MJ Rangarajan S McQueen MJ Poirier P Wielgosz A Association of urinary sodium and potassium excretion with blood pressure. N Engl J Med. (2014) 371(7):601–11. 10.1056/NEJMoa1311989 25119606

[44] Grillo A Salvi L Coruzzi P Salvi P Parati G. Sodium intake and hypertension. Nutrients. (2019) 11(9):1970. 10.3390/nu11091970 31438636 PMC6770596

[45] Ge Z Zhang J Chen X Yan L Guo X Lu Z Are 24 h urinary sodium excretion and sodium:potassium independently associated with obesity in Chinese adults? Public Health Nutr. (2016) 19(6):1074–80. 10.1017/s136898001500230x 26228639 PMC10271176

[46] Yin L Mei J Dong J Qu X Jiang Y. Association of sodium intake with adverse left atrial function and left atrioventricular coupling in Chinese. J Hypertens. (2023) 41(1):159–70. 10.1097/hjh.0000000000003317 36453659 PMC9794161

[47] Young MJ Rickard AJ. Mechanisms of mineralocorticoid salt-induced hypertension and cardiac fibrosis. Mol Cell Endocrinol. (2012) 350(2):248–55. 10.1016/j.mce.2011.09.008 21930186

[48] Gdowski MA Murthy VL Doering M Monroy-Gonzalez AG Slart R Brown DL. Association of isolated coronary microvascular dysfunction with mortality and major adverse cardiac events: a systematic review and meta-analysis of aggregate data. J Am Heart Assoc. (2020) 9(9):e014954. 10.1161/jaha.119.014954 32345133 PMC7428565

[49] Tran TV Djaileb L Riou L Lantuejoul LR Giai J Barone-Rochette G. Coronary microvascular dysfunction as assessed by multimodal diagnostic imaging in patients with hypertrophic cardiomyopathy is related to the severity of cardiac dysfunction. Microcirculation. (2024) 31(2):e12843. 10.1111/micc.12843 38174616

[50] Vîjîiac A Scărlătescu AI Petre IG Vîjîiac C Vătășescu RG. Three-dimensional combined atrioventricular coupling index-A novel prognostic marker in dilated cardiomyopathy. Biomedicines. (2024) 12(2):302. 10.3390/biomedicines12020302 38397904 PMC10886977

[51] Wen J Tu J Tao X Tang Y Yang Z Pan Z Cardiac magnetic resonance left atrioventricular coupling index as a prognostic tool in hypertrophic cardiomyopathy. ESC Heart Fail. (2025). 10.1002/ehf2.15237 39905775 PMC12055398

[52] Fan J Wang H Ma C Zhou B. Characteristics of atrial ventricular coupling and left atrial function impairment in early Fabry disease patients using two-dimensional speckle tracking echocardiography. Int J Cardiol. (2025) 422:132967. 10.1016/j.ijcard.2025.132967 39814185

[53] Meng F Li J Zhao R Wu Y Liu Y Yang Y Left atrioventricular coupling index assessed with three-dimensional echocardiography: a prognostic marker of short-term outcomes in light-chain cardiac amyloidosis. Amyloid. (2025) 32:63–71. 10.1080/13506129.2024.2448435 39815461

[54] Wang Y Bi K Wan K Liu J He W Li X Cardiovascular magnetic resonance-derived left atrioventricular coupling index as a novel prognostic marker for light-chain amyloidosis. Int J Cardiol. (2025) 418:132630. 10.1016/j.ijcard.2024.132630 39395718

[55] Chen Y Zhang N Zhao W Sun Z Liu J Liu D Incremental prognostic value of left atrial strain in patients with suspected myocarditis and preserved left ventricular ejection fraction. J Magn Reson Imaging. (2025) 61(2):899–908. 10.1002/jmri.29429 38722216

[56] Nishimura RA Otto CM Bonow RO Carabello BA Erwin JP 3rd Fleisher LA 2017 AHA/ACC focused update of the 2014 AHA/ACC guideline for the management of patients with valvular heart disease: a report of the American college of cardiology/American heart association task force on clinical practice guidelines. J Am Coll Cardiol. (2017) 70(2):252–89. 10.1016/j.jacc.2017.03.011 28315732

[57] Zsarnoczay E Varga-Szemes A Schoepf UJ Rapaka S Pinos D Aquino GJ Predicting mortality after transcatheter aortic valve replacement using AI-based fully automated left atrioventricular coupling index. J Cardiovasc Comput Tomogr. (2025):S1934-5925(24)00582-3. 10.1016/j.jcct.2024.12.082 39794233

[58] Pezel T Michos ED Varadarajan V Shabani M Venkatesh BA Vaidya D Prognostic value of a left atrioventricular coupling index in pre- and post-menopausal women from the multi-ethnic study of atherosclerosis. Front Cardiovasc Med. (2022) 9:1066849. 10.3389/fcvm.2022.1066849 36479563 PMC9719991

[59] Pezel T Venkatesh BA Vasconcellos HD Kato Y Post WS Wu CO Determinants of left atrioventricular coupling index: the multi-ethnic study of atherosclerosis (MESA). Arch Cardiovasc Dis. (2022) 115(8–9):414–25. 10.1016/j.acvd.2022.04.011 35906156

